# Supplementary figures and images for: Systematic Functional Annotation Workflow for Insects
Source: Insects. 2022 Jun 27;13(7):586. doi: 10.3390/insects13070586 (PMC9319598; doi:10.3390/insects13070586)

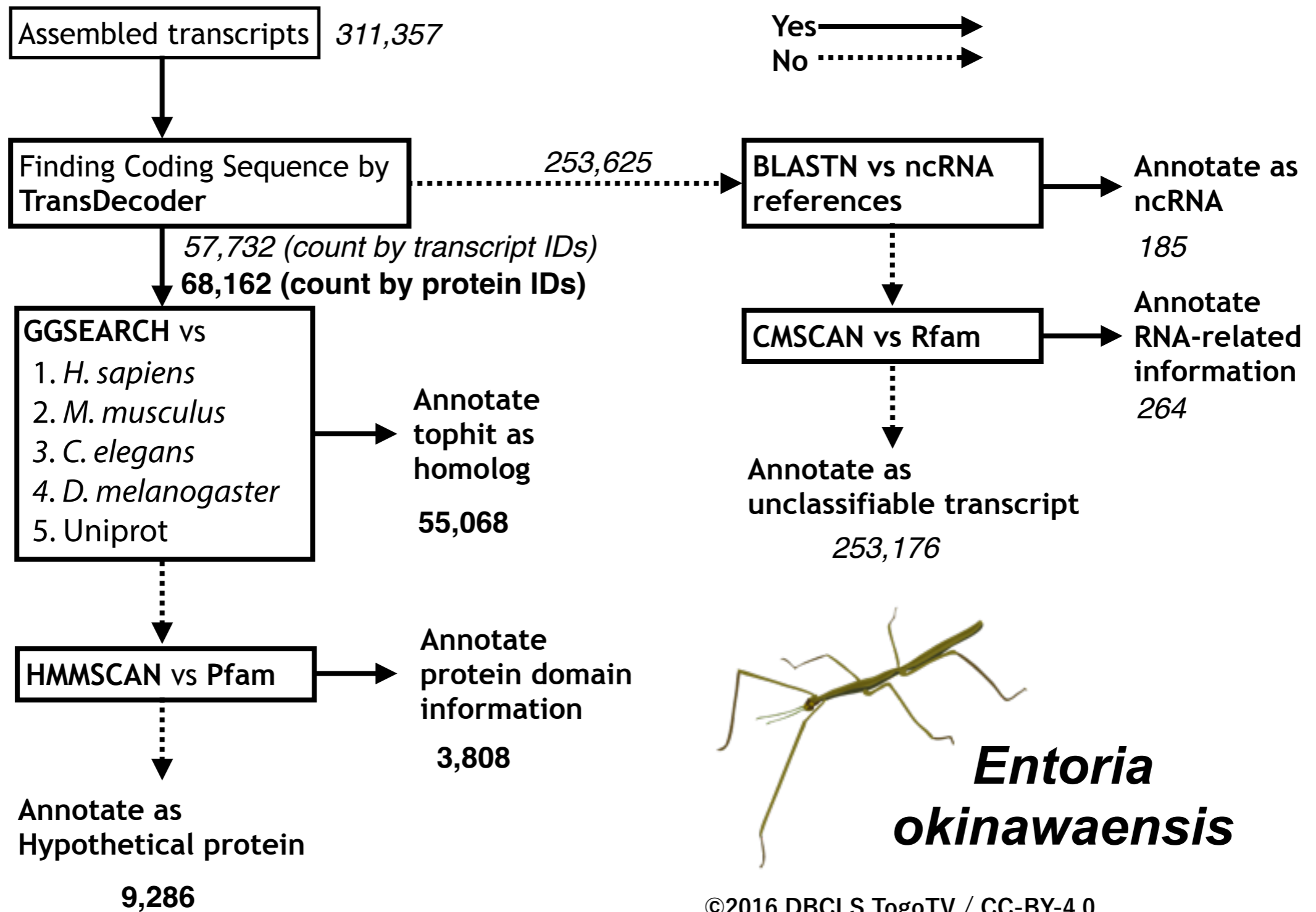

**Figure S1**

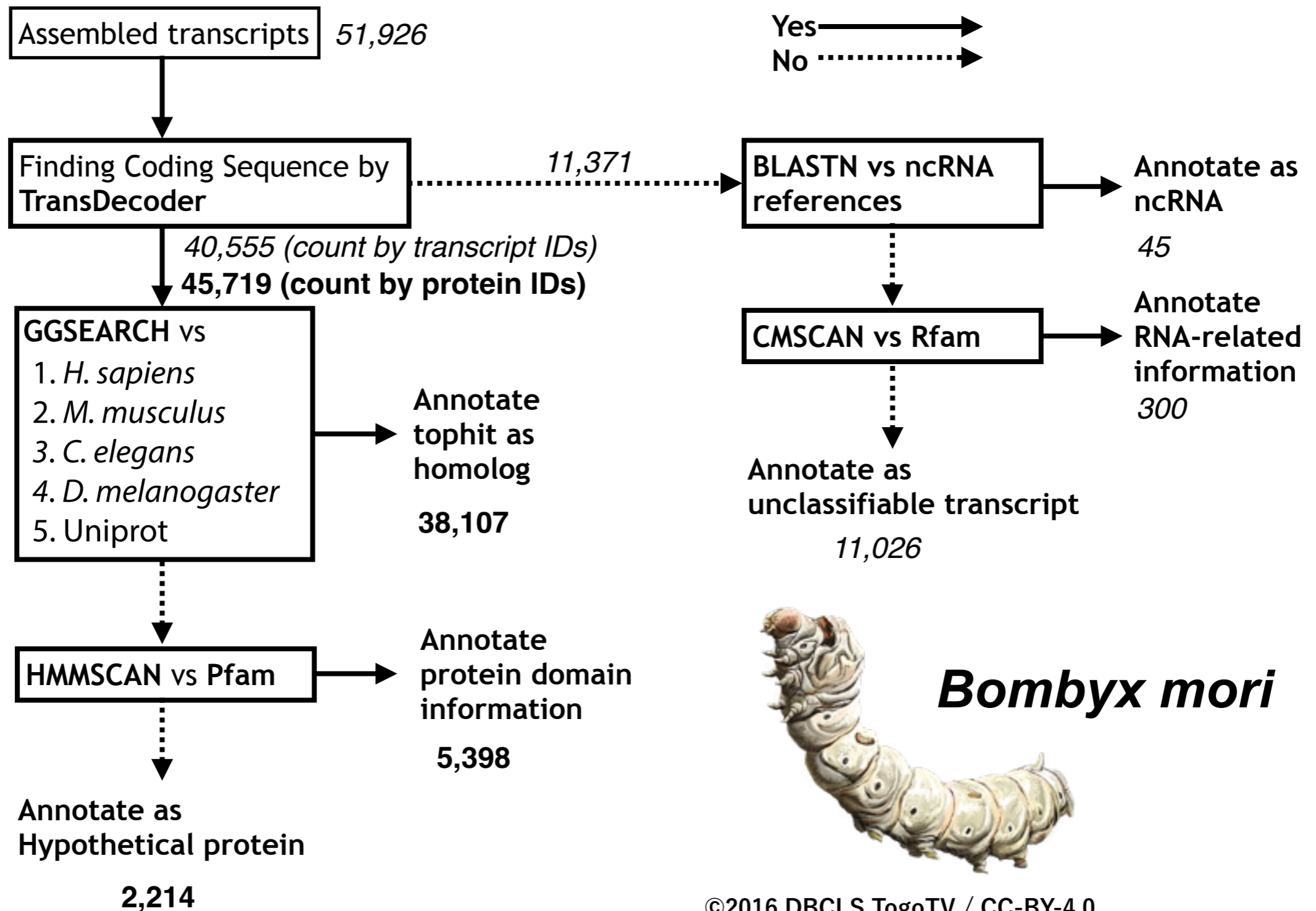

**Figure S2**

Supplement: Supplementary file 1 [file insects-13-00586-s001.zip › insects-1763735-supplementary.pdf]
